# An openEHR based infection control system to support monitoring of nosocomial bacterial clusters and contacts

**DOI:** 10.1038/s41746-025-01795-9

**Published:** 2025-06-30

**Authors:** Pascal Biermann, Claas Baier, Ann Christin Vietor, Benedikt Zacher, Tom Baumgartl, Tatiana von Landesberger, Michael Behnke, Michael Storck, Markus Petzold, Martin Kaase, HiGHmed lnfection Control Study Group, Pascal Biermann, Claas Baier, Benedikt Zacher, Tom Baumgartl, Michael Behnke, Michael Storck, Martin Kaase, Sarah Ballout, Tim Eckmanns, Petra Gastmeier, Julian Varghese, Vanessa M. Eichel, Nicolas Reinoso-Schiller, Sabine Hanß, Tibor Kesztyüs, Alexander Dalpke, Max Klass, Angela Merzweiler, Jennifer Daniela Elke Hoos, Peter Brunnecker, Alexander Mellmann, Hauke Tönnies, Cora Drenkhahn, Benjamin Gebel, Joshua Wiedekopf, Tatiana von Landesberger, Dirk Schlüter, Michael Marschollek, Simone Scheithauer, Antje Wulff, Dirk Schlüter, Michael Marschollek, Simone Scheithauer, Antje Wulff

**Affiliations:** 1 Peter L. Reichertz Institute for Medical Informatics of TU Braunschweig and Hannover Medical School, Hannover Medical School, Hannover, Germany; 2 Big Data in Medicine, Carl von Ossietzky Universität Oldenburg, Oldenburg, Germany; 3 Institute for Medical Microbiology and Hospital Epidemiology, Hannover Medical School, Hannover, Germany; 4 Robert Koch Institute, Berlin, Germany; 5 University of Cologne, Cologne, Germany; 6 Charité-Universitätsmedizin Berlin, corporate member of Freie Universität Berlin, Humboldt-Universität zu Berlin, and Berlin Institute of Health, Institute of Hygiene and Environmental Medicine, National Reference Centre for Surveillance of Nosocomial Infections, Berlin, Germany; 7 Institute of Medical Informatics, University of Münster, Münster, Germany; 8 Institute of Medical Microbiology and Virology, University Hospital ‘Carl Gustav Carus’, University of Technology Dresden, Dresden, Germany; 9 Department of Infectious Diseases, Medical Microbiology and Hygiene, Heidelberg University Hospital, Heidelberg, Germany; 10 Department for Infection Control and Infectious Diseases, University Medical Centre Göttingen (UMG), Georg-August University Göttingen, Göttingen, Germany; 11 Institute of Medical Informatics, University Medical Center Göttingen (UMG), Georg-August University Göttingen, Göttingen, Germany; 12 University Hospital Heidelberg, Heidelberg, Germany; 13 Institute of Medical Informatics, Heidelberg University Hospital, Heidelberg, Germany; 14 Berlin Institute of Health at Charité – Universitätsmedizin Berlin, Core Facility IT, Charitéplatz 1, 10117 Berlin, Germany; 15 Charité – Universitätsmedizin Berlin, Medizinisches Datenintegrationszentrum, Charitéplatz 1, 10117 Berlin, Germany; 16 Institute of Hygiene, University Hospital Münster, Münster, Germany; 17 IT Center for Clinical Research (ITCR-L) and Institute of Medical Informatics (IMI), University of Lübeck, Lübeck, Germany; 18 Department of Infectious Diseases and Microbiology, University Medical Center Schleswig-Holstein, Lübeck, Germany

**Keywords:** Infectious-disease diagnostics, Antimicrobial resistance, Infectious diseases, Software, Standards, Data integration

## Abstract

Early outbreak detection, allowing rapid intervention, is essential to reduce the burden of healthcare-associated pathogen transmission, including multidrug-resistant bacteria. Digital, routine data-driven solutions are promising, but often proprietary, non-interoperable, or limited in functional scope. The open-source Smart Infection Control System (SmICS) offers automatic calculations and interactive views on patients' movement and lab data, epidemic curves, contact networks, complemented by temporal-spatial visualizations. It is an open-source software based on openEHR as an interoperability standard and was evaluated by assessing time efficiencies in performing basic infection control tasks (e.g., contact networks) and usability with the System Usability Scale (SUS). Evaluated at three sites, SmICS reduced the time needed for performing routine infection control tasks by up to 81.47% (68.5 min (95%CI [30.5–106.5])) reaching a SUS of 51.6 points. The study reveals time savings through the use of SmICS in daily tasks, but also identified usability issues and a need for minimizing query waiting times.

## Introduction

Nosocomial outbreaks induced by transmission of viruses, susceptible and multi-drug resistant bacteria (MDRB) or fungi^1–3^ are a major challenge in infection prevention and control (IPC). Outbreaks often have a negative impact on patients, patient care and the affected institution^4^. Patient outcomes are worsened and healthcare costs are increased when outbreaks lead to a significant surge of hospital-acquired infections^5,6^. Even the frequent occurrence of bacterial colonization with a clonal strain in multiple patients (known as colonization clusters) is considered problematic from an IPC perspective. Moreover, nosocomial transmission of MDRBs contributes to the spread of multidrug resistance^7^.

Early detection and proactive control are essential as they can limit the scale of the outbreak by allowing countermeasures to be taken quickly. Nowadays, outbreak analysis in hospitals often relies on time-consuming manual work by IPC teams (IPCT), comprising a comprehensive analysis of patients' movement data and microbiological test results. Required data is often extracted manually from disparate, heterogeneous primary software sources. This manual approach is error-prone and protracted^8^. Additionally, the consecutive analysis of available data varies from person to person if performed in a non-standardized manner. Considering the increasing and complex workload of IPCTs (for instance during the COVID-19 pandemic^9^), it seems necessary to make IPCT daily tasks as efficient, comprehensive, objective, simple, and reliable as possible.

To reduce the manual workload of IPCTs, digital solutions can be used to automatically calculate contact networks or analyze transmissions, supporting the detection of clusters of pathogens potentially reflecting a beginning outbreak. The freed resources can then be used to focus on verifying and analyzing the outbreak and initiating interventions. The current situation in healthcare is very promising, as the increasing digitalization in medical documentation entails a growing pool of invaluable data, also for IPC^10^. Today, a vast amount of data about patients is available in a digital, machine-readable format, thus allowing for automated processing. Moreover, different digital approaches for outbreak detection at different levels already have been developed, tested and reported^11–14^. Solutions have been established against the backdrop of the COVID-19 pandemic^15^. A review by Swaan et al. concludes that computerized systems reduce delay in reporting and that timely detection is crucial for outbreak containment^16^.

However, most automated detection systems rely on institution-specific database formats or are commercial, vendor-dependent software systems that are not freely available. These systems often lack standardized data definitions and openly accessible application programming interfaces (APIs), making it a challenge to connect them in a timely manner and with proper data quality, in terms of semantics and harmonization, to the current hospital information system (HIS). The HIS landscape contains various, disparate and heterogeneous proprietary applications holding an institution’s relevant routine data in non-standardized data formats^17,18^. Syntactically accessing routine and research data from these “data silos” is demanding, and understanding the original meaning of the data as intended (in terms of semantic interoperability) is even more difficult. However, this is a prerequisite for the development of intelligent, decision-support applications based on routine data. Data and algorithms are often not accessible for secondary uses and analysis or for further adjustment, enhancement or optimization. Additionally, a comprehensive IT solution for IPC should be designed to work across institutions at regional, national, and international levels, considering that healthcare providers, such as hospitals in a regional network, frequently transfer patients. To overcome these barriers, by relying on the interoperability standard openEHR, the HiGHmed Infection Control project aims to develop an open-source, standards-based and interoperable application to support IPCTs in their daily work^19–21^. In previous work, we have already taken the first step towards standardization: we have identified openEHR-based data models that can be used to transform infectious disease-related primary source data into internationally agreed-upon data representations without loss of information and quality, thus forming the basis of our intended application^22^.

In this paper, we report the design, implementation and evaluation of an open-source, openEHR-based software application for automated and time-efficient monitoring, contact networks and epidemiologic analysis to support infection control specialists in detecting nosocomial bacterial clusters, including MDRB, during their daily work in hospitals, called Smart Infection Control System (SmICS).

## Results

### SmICS—Smart Infection Control System

SmICS is an open-source, openEHR-based application for supporting IPCTs in infection control tasks such as monitoring and detection of suspected nosocomial bacterial transmission events, including clusters and outbreaks and for analyzing contact networks. The software package, together with an exemplary openEHR-based data set, is freely available on GitHub^23,24^.

**Fig. 1:**
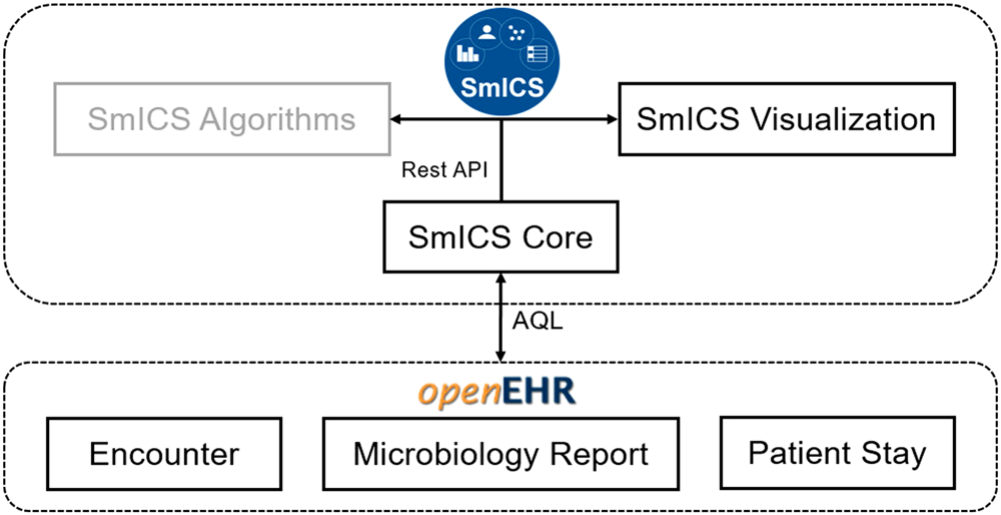
SmICS architecture. The underlying openEHR data models are available at: Encounter [https://ckm.highmed.org/ckm/templates/1246.169.620], Microbiology Report [https://ckm.highmed.org/ckm/templates/1246.169.69/46], Patient Stay [https://ckm.highmed.org/ckm/templates/1246.169.620].

The SmICS architecture is presented in Fig. 1. SmICS is a web application consisting of separated services. “SmICS Core” offers functionalities for tabular views summarizing information about a certain patient, a ward and spatial-temporal contacts between patients. It also acts as a data pre-processor unit for the second component, a Node.js application as open-source JavaScript runtime environment, called “SmICS Visualization”. It provides rich interactive visualization interfaces and enhanced temporal-spatial graphics. The integration of a third component “SmICS Algorithms” is currently in development. Connection to the underlying openEHR data repository is fully standard-based by using a RESTful API and the Archetype Query Language. Participating sites filled this repository with data at their sites using standardized agreed data models (openly available at the Clinical Knowledge Manager, see Fig. 1); no local and site-specific databases are accessed.

**Fig. 2:**
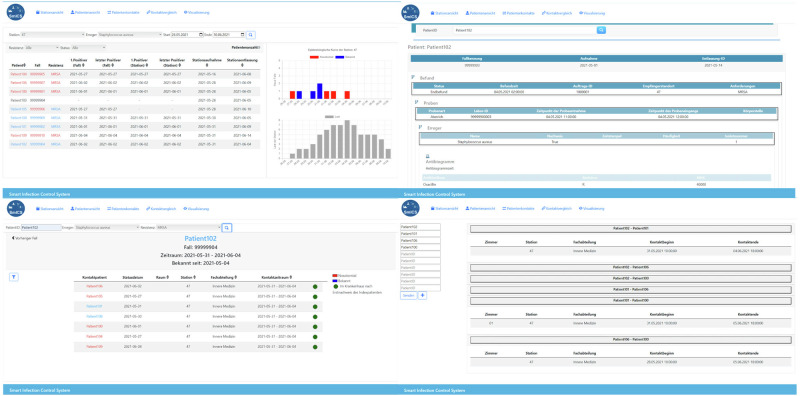
SmICS Core. Ward Overview (top left), Patient View (top right), Contact Network (bottom left), Contact Comparison (bottom right).

*SmICS Core* offers four main functionalities (see Fig. 2): a ward overview, a patient view, a contact network view, and a contact comparison.

- The “Ward Overview” presents data on a specific ward, focusing on a specific pathogen (e.g., a specific bacterial species) over a specified time period, including details of patients who visited the ward and the calculated acquisition status for the target pathogen (hospital versus community-acquired). In addition, the results view is designed to provide information on resistance, where applicable (e.g., methicillin resistance in *Staphylococcus aureus*). To calculate the acquisition status, the given rules from the domain experts were integrated. If a pathogen had a positive microbiology result within the first two days of the encounter, the pathogen is considered “community-acquired”. If a pathogen showed a positive microbiology result on day three or later of the hospital stay, it was considered “hospital-acquired”, unless the pathogen had been previously identified in the patient’s medical history. Within the status “hospital-acquired”, a distinction can be made whether an acquisition was made on the same or another ward. It also produces two epidemiological graphs revealing the number of new and all cases on the ward. The Ward Overview feature was applied in task 1 of the efficiency evaluation.
- The “Patient View” displays all microbiology results associated with a patient’s hospital stay and provides subdivided sections to adjust the level of detail displayed.
- The “Contact Network” computes based on the “Encounter” and the “Patient Stay” data the contacts (co-location on the same ward or room, respectively) and lists, the contact location and the contact time for a patient during the hospital stay. The co-location on the same ward or room does not have to be contemporaneous; rather, it is sufficient for the two events to occur within a 24-h time slot. To obtain further information on the similarity of pathogen findings, the relevant MDRB of interest must be selected. The respective patients are colored if a positive MDRB result is available.
- The “Contact Comparison” allows two or more patients to be entered. SmICS calculates

whether the patients have had any temporal-spatial contact.

**Fig. 3:**
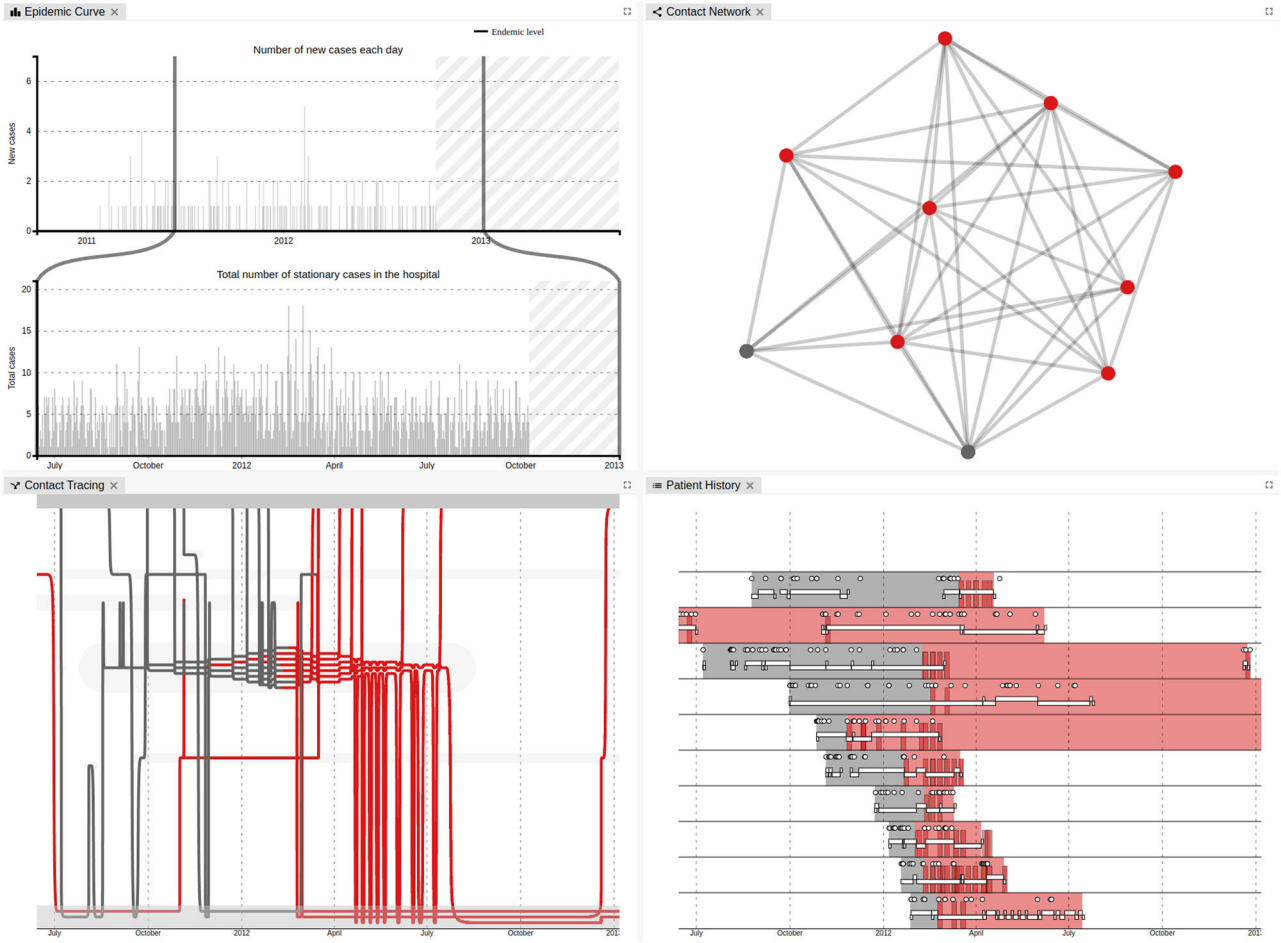
SmICS Visualization. Epidemic Curve (top left), Contact Network (top right), Contact Tracing (bottom left) and Patient History (bottom right) offer distinct perspectives, allowing users to interactively analyze patient data. All four visualizations support interactive actions. Mouse-overs provide additional patient information, detailed information on contact duration and location, or microbiological test results as tooltips for each visual element. Left-clicking on visual elements of patients enables filtering them, aiding visual analysis by making filtered elements opaque. Furthermore, all three timeline visualizations—Epidemic Curve, Contact Tracing and Patient History—offer mouse interactions to change the displayed time frame. Any changes to patient and time filter settings are simultaneously applied across all open views.

*SmICS Visualization* offers four different views (see Fig. 3):

- The “Epidemic View”^25^ consists of two bar charts on a timeline. The lower bar shows the total number of patients with a pathogen of interest in the hospital, while the upper bar shows the number of new patients with that pathogen. This interactive visualization allows users to gain insight into the known epidemic situation and to interactively narrow down the time period to be analyzed.
- The “Contact Network”^25^ represents patients as circles and contacts between two patients are represented by linking lines. A contact is defined as co-location of patients on the same ward or room, respectively (not necessarily contemporaneous, see above). The color of the circle indicates the result of a microbiology report: gray represents unknown, whereas red represents a positive result. This view enables users to analyze the underlying event-based dynamic graph as a whole. It provides information on which patients had contact, assisting in the identification of patient clusters.
- The “Contact Tracing”^26^ is a left-to-right timeline visualization where patients are depicted as lines, and their status with regard to pathogen positivity is represented by the line color. Similar to the “Contact Network”, gray represents an unknown status, whereas red represents a positive status. The vertical position of the lines indicates the patient’s location within the facility. When a patient changes wards, their line shifts vertically at that time, reflecting the change in position. If multiple patients are on the same ward, their lines are bundled closely together, indicating contact. This visualization allows users to observe the sequence and temporal spacing of infections, contacts and ward changes, associating them with an outbreak.
- The “Patient History”^26^ lists patients in a vertical sequence (left-to-right timeline). The patient’s background color represents their microbiological status from the first admission to the last discharge. Similar to the other views, gray represents an unknown status, whereas red represents a positive status. Ward stays are represented by horizontal rectangles, microbiological test results as vertical rectangles, and other medical or nursing procedures (e.g., dressing change) as dots.

### Efficiency and usability evaluation

**Fig. 4:**
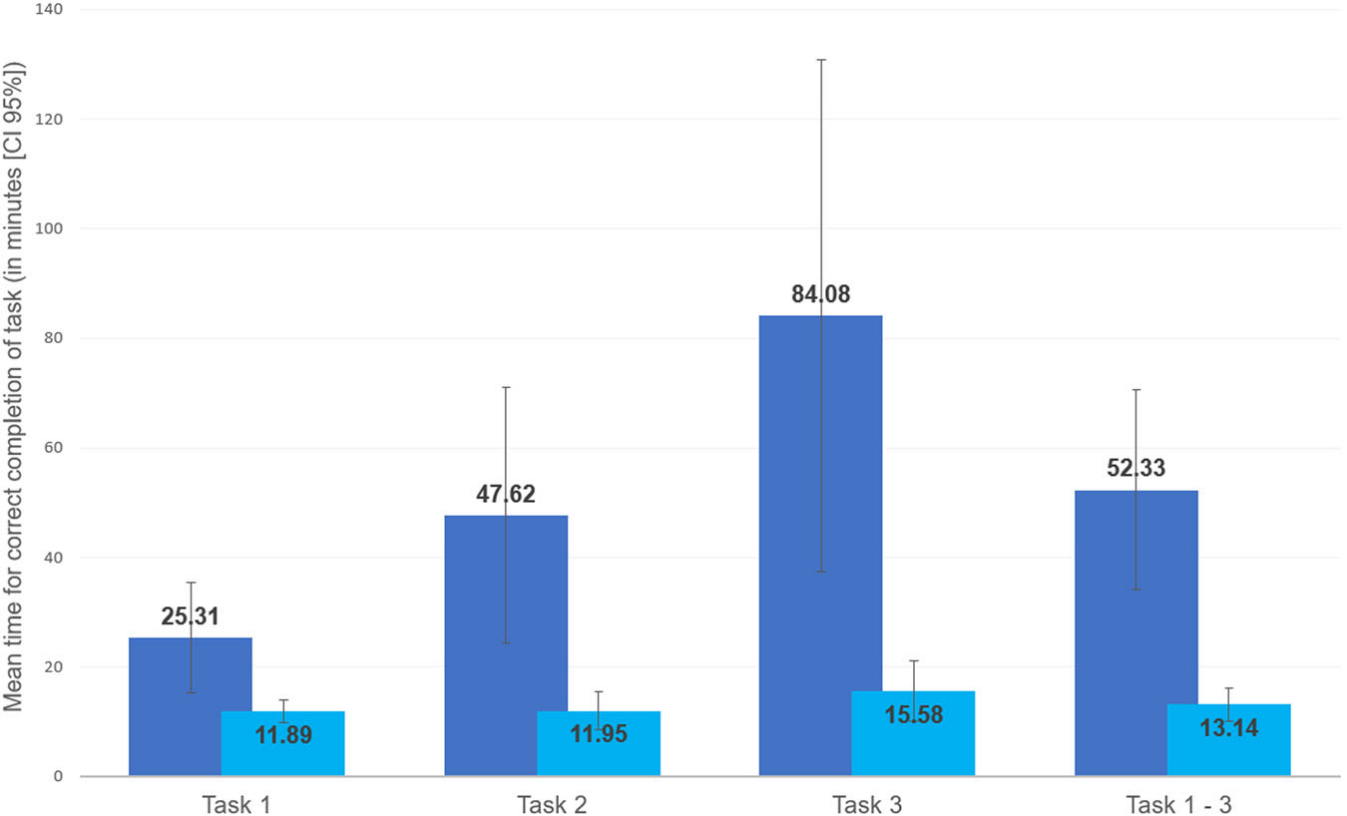
Comparison of time efficiency in performing exemplary tasks of IPC work in a standard procedure versus using SmICS. Results of SmICS efficiency evaluation. Comparison of time efficiency (required time for correct completion of task) in performing exemplary tasks of IPC work in a standard procedure (blue) versus using SmICS (light blue) at three partner sites.

Three university hospital partner sites implemented SmICS on routine real data and were fully able to participate. Figure 4 represents the results of the time measurements. In eight of nine task/site combinations, the time required for correct completion of the tasks was shorter when using SmICS. The amount of time saved in average varied depending on the task. At the lower end, the first task saved in average 13.4 min (95%CI [5.0–21.9]), which led to a total time saving of 53.00%. The best result was achieved in the third task, which led to an average of 68.5 min (95%CI [30.5–106.5]), saved or a total of 81.47%. Overall, the SmICS led to an average time saving per task of 39.2 min (95%CI [24.0–54.4]), which is a total of 74.89%. A detailed evaluation can be found in the supplementary information.

**Fig. 5:**
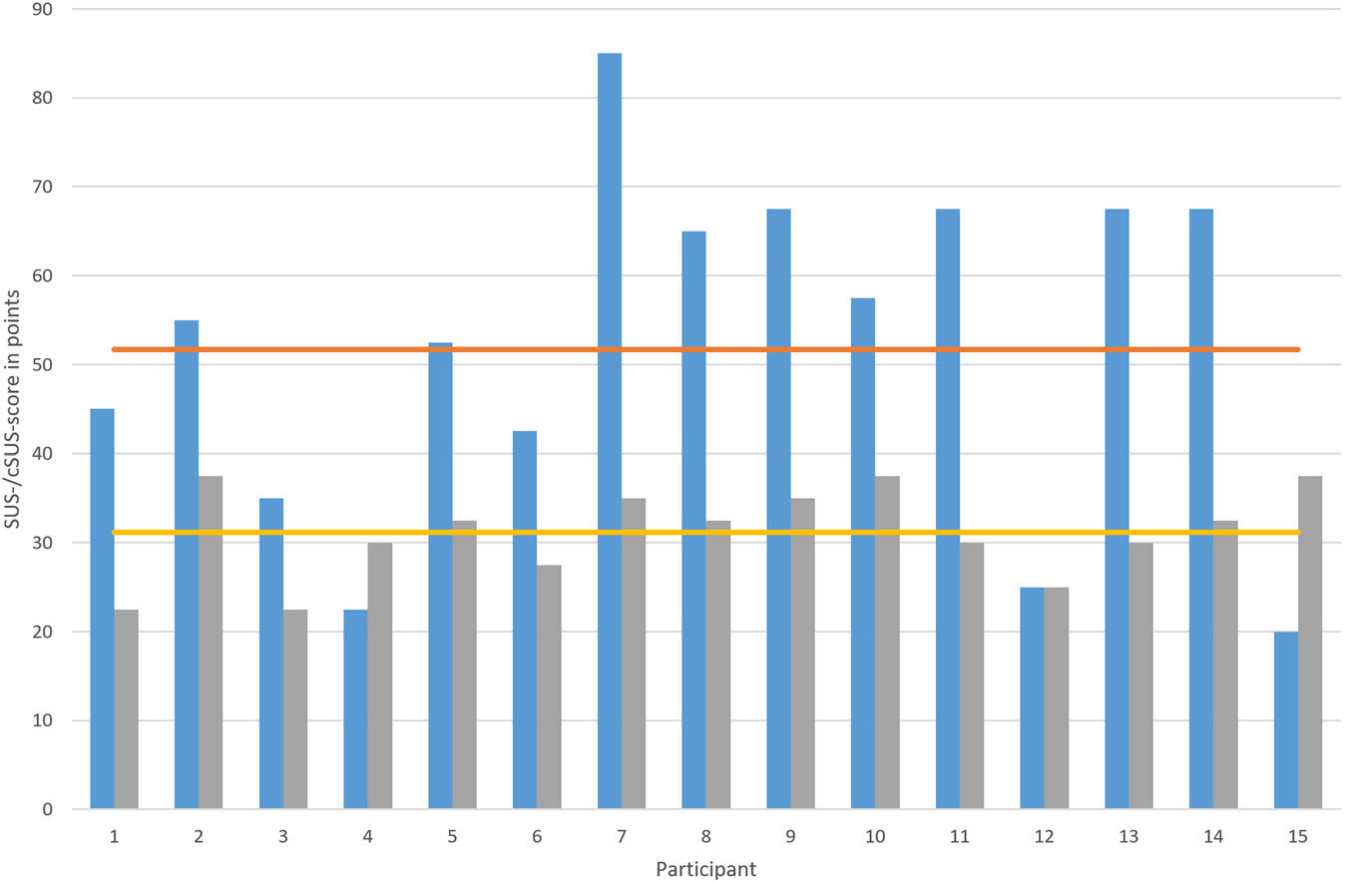
SUS- and cSUS-scores of evaluation participants. SUS-score (blue), cSUS-score (gray), SUS average (orange) and cSUS average (yellow) results of SmICS usability evaluation with 15 participants from three partner sites.

**Fig. 6:**
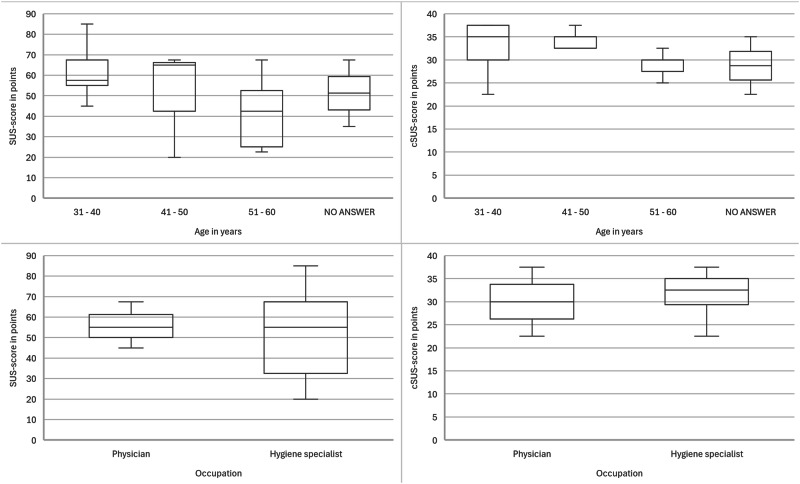
SUS- and cSUS-scores of the evaluation focused on age and occupation. SUS-score evaluation concerning age groups (top left), cSUS-score concerning age groups (top right), SUS-score concerning occupation (bottom left) and cSUS-score concerning occupation (bottom right).

Each of the three sites that implemented SmICS also participated in the usability survey with at least five IPC experts (12 hygiene specialists and 3 physicians). For evaluation purpose, the System Usability Scale (SUS) and the Clinical System Usability Scale (cSUS) score were used. These are scores for measuring usability of new software systems in general and in clinical settings. The SUS ranges from 0 to 100 points with 100 points resulting in a prefect usability^27^. Same applies for the cSUS, except that the maximum value is 50. The evaluated SUS ranges from 20 to 85 points, 51.6 on average. The cSUS ranges from 22.5 to 37.5 points, 31.1 on average (see Fig. 5). Figure 6 shows that the age of the participants has an influence on the usability rating. The eldest participant group has been observed to rate the lowest usability. However, the profession of the participants has no influence on the usability evaluation.

## Discussion

In this work, we present SmICS as a digital supporting tool for infection control tasks such as monitoring of bacteria, contact networks and epidemiological analysis. Nosocomial outbreaks caused by different bacterial and viral pathogens, e.g., MDRBs^28^ or the severe acute respiratory syndrome coronavirus 2, are one of the major challenges in IPC^29^. Depending on the kind of pathogen and source, transmission dynamics vary greatly, necessitating a broad and deep IPC expertise in timely recognition to implement the best suitable intervention measures aiming at reducing the spread and controlling the outbreak. Smart software solutions which support IPCTs in their daily work are very promising and can help them to anticipate and better understand potential transmission chains in the light of massive amounts of available data^30^. Tasks such as contact tracing and sophisticated epidemiological analysis of MDRBs at the ward level are particularly time-consuming when using the traditional manual approach. Especially against the background of the shortage of skilled labor and the difficult recruitment of new IPCT members, digital supporting systems that release time capacities of IPCTs needed for verification of transmission events, identifying potential sources, choosing the most appropriate intervention strategies and finally evaluating their success are of special value. This is particularly true in times of pandemic, which dramatically highlights the increasing workload pressures in the healthcare sector^31^. Our digital solution to this challenge, SmICS, provides multiple functions for efficient support in routine infection control tasks. For this purpose, microbiological data and movement data are combined as essential data sources and automatically analyzed in a suitable manner. Using historical, real-world data sets, the functionality of SmICS was successfully evaluated on the basis of three practical IPC tasks. Our analysis showed that (depending on the site and the tools available locally from routine care), by using SmICS, routine infection control tasks can be carried out efficiently, resulting in an average time saving of 39.2 min (95%CI [24.0-54.4]) for all defined tasks and individual savings of up to 68.5 min (95%CI [30.5-106.5]) for one specific task. All participants were able to complete the tasks without errors.

Furthermore, SmICS is fully openEHR-based and provides open interfaces to allow secondary use of data, enhancement of algorithms, sharing of results and reuse of the application across institutions. The use of openEHR templates, reviewed and accepted by domain experts, helps to provide information about which data is required for the flawless use of the SmICS, e.g., by using cardinalities on an archetype and element level. On top of that, openEHR models permit automated constraint and plausibility checks during the storage of data, thereby ensuring syntactic and semantic correctness to a higher extent. Nonetheless, the quality and completeness of data captured in routine clinical settings can vary considerably, influenced by differences in documentation practices, data entry errors, and incomplete records. There still is the risk that such variability may affect the reliability, validity, and generalizability of real-world data-based tools. Since we fully rely on openEHR-based data, no such technical variability has been seen in our SmICS evaluation. To evaluate data quality and variability effects, an enhanced analysis of, on the one hand, the extraction, transformation and loading processes in the medical data integration centers (MeDICs) and, on the other, the original documentation processes are potential mitigation strategies. Furthermore, given that our SmICS is conceptualized as a fully routine data-based approach, it is important to note that utilization is constrained to the available data. For instance, data pertaining to healthcare workers may be of significant interest in the context of infection control. However, the collection of the requisite data with the necessary level of detail for such purposes (e.g., by means of tracking devices) is subject to strict legal regulations, thus, such data is not widely available in routine data sets. In addition, more extensive forms of contact tracing—such as those conducted during the COVID-19 pandemic, which also included non-hospital settings (e.g., nursing homes)—are currently not implemented in SmICS. If data on patient presence in functional areas are available in the primary data systems (e.g., as part of the movement data), they could, in principle, be integrated into SmICS.

The interoperable and openEHR-based design of SmICS has enabled us to roll out the application in a timely manner to various German university hospitals with different primary source system infrastructure landscapes. Our interoperable concept makes future cross-location analysis and sharing concepts possible, which will become particularly important in an increasingly interconnected healthcare system that calls for quick collection and analysis of data, particularly in times of crisis.

Alleviating the burden of daily IPC workload by means of innovative digital solutions has been the subject of research before. In 2024, Arzilli et al. published a scoping review of articles from 2018 to 2023, presenting an overview of new practical technologies to support traditional infection control and surveillance work in real settings. The use of different health informatics technologies, natural language processing, digital health/e-health/m-health applications, mobile computing, and the reuse of electronic health record data has been recently investigated, with machine learning-based approaches emerging as the predominant area of research^32^. Related, the authors also acknowledge the need for both in-depth expertise and large volumes of sensitive data to be available in order to be able to train and apply these machine learning-based approaches in real settings. Moreover, the majority of research work focuses on the timely detection of specific infections or general surveillance statistics^32^. Digital, low-barrier, routine data-based approaches for infection control support in multidrug-resistant bacteria as in the sense of digital contact networks or tracing, as anticipated by SmICS, are scarce. One related example, however, focusing specifically on the retrospective contact network analysis of Vancomycin-resistant Enterococcus transmissions, has been researched by Neumann et al. in 2020^33^. They point out the advantages of infectious-contact networks and the potentials of also considering negatively tested patients to reveal intra-hospital pathways of spreading and to support infection prevention planning^33^. Although delivering a visual representation for analysis, their objective has not been to implement a practical application, as was achieved with SmICS. However, it should be noted that Neumann et al. further incorporated genomic analyzes, which could prove to be a valuable prospective feature to be integrated into SmICS.

Obviously, in the Corona pandemic, related approaches focusing virologic pathogens have emerged. For example, in the Hospital Clínic de Barcelona, a surveillance system called CoSy-19, was developed to interrupt intra-hospital transmission of infections and enabling timely implementation of interventions, by using contract tracing and epidemic curves, amongst other^34^. Another line of research of significant importance within the domain of infection control pertains to the detection of clusters and outbreaks, a function not yet integrated into SmICS. In 2009, the Brigham & Woman’s Hospital in Boston, Massachusetts, conducted a retrospective study on microbiology data from 2002 to 2006 with a tool called WHONET-SaTScan. This is an automated cluster detection tool based on two freely available software packages: SaTScan, a software developed for geographical disease surveillance, and the WHONET/BacLink, a software for descriptive analysis of microbiology data^13^. Another exemplary cluster alert system, named CLAR, was implemented in the Charité, a tertiary care hospital in Germany. This system uses six algorithms for detecting potential outbreaks on a ward. In 2019, a prospective study was conducted, in which the system generated automated alerts, 35% of which were classified as relevant alerts requiring further investigation^30^.

In contrary to the presented related work, the idea of SmICS is to have a software that serves as an IPC program fully operating on an interoperability standard. To the authors’ knowledge, utilization of clinical routine data in a digital application through an interoperable, standardized approach with interactive visualization interfaces that is not restricted to a specific pathogen is a distinctive feature of the present study.

SmICS was fully implemented and evaluated at only three of the participating sites. The technical implementation proved to be challenging because there were difficulties in integrating microbiological findings and movement data into the local MeDICs due to strict data protection issues or limited access to proprietary primary clinical systems. An identified issue with SmICS is its technical performance, resulting in long waiting times for the users and, thus, dissatisfaction of the users with the system’s usability. These waiting times depended on the amount of data which were stored within the MeDIC data repository and how many resources were allocated to the data repository, in the form of RAM and CPU, and if the data repository was set up directly or by using a dockerized version. Due to the different infrastructural strategies of each participating MeDIC, waiting times were not considered in the evaluation, as they were not due to the waiting times of the core software, but to delays caused by the underlying MeDIC data repository. Prior to the start of the study, it was not recognized that waiting times would exert such a significant effect across all sites. Consequently, the study protocol did not include the measurement of delays, thus constituting a limitation in study design. Meanwhile, the MeDIC infrastructures underwent changes, so it is not possible to reproduce the waiting times. Basically, it is important to note that there are simply a lot of data points to process, resulting in long query response times. These loading problems have also been reported in other related work (such as the WHONET-SaTScan). Of course, data integration optimizations or the inclusion of further indexing structures are currently underway to solve this major issue. For example, the new version of EHRbase, which is an open-source database implementation of openEHR, now allows indexing at the level of each data element path, which can result in improved query performance^35^. Another solution could be to overcome live querying by implementing a user-modifiable daily morning batch load, as reported in Stachel et al.^14^.

These waiting times also seem to be the main reason for the System Usability Score of 51.6 (“OK' according to Bangor et al.^27^). 14 out of 15 participants scored the SUS lower than 71.4 points (“good” according to Bangor et al.^27^). It is evident that SmICS, in light of the technical issues that have been identified, did not attain a satisfactory level of usability. The performance issues were also mentioned in the answers to the open-ended questions. Additionally, in contrast to the quantitative time efficiency results, subjective usability-based feeling of time-efficiency was rather low: the second statement of the cSUS (“Effective support for this software is hard to access in a clinically-appropriate timescale”) resulted in an average of 3.5 (5 referring to “Agree strongly”, meaning not time efficient). However, it has to be mentioned that contact tracing with extensive movement data and huge amounts of lab data in hospitals with ~60,000 inpatients per year is a data-intensive, demanding task, and—considering the limited human IPCT resources—may be even impossible without computational support.

The technical platform of SmICS offers the possibility of developing into an advanced, interoperable, open-source infection control assistance suite. Important functions such as contact chains and the tracing of potential transmission events—to be verified or falsified by the IPC team later on—and the performance of smaller routine epidemiological tasks (for instance creating a ward-based epidemiological curve for a specific pathogen based on raw data not verified to belong to the event yet) are integrated. Another important task that IPCTs are involved in on an ongoing basis for a significant proportion of their working time is surveillance for certain bacterial and viral species, sometimes based on resistance patterns. This is a legal requirement for hospitals in many countries, including Germany (German Infection Protection Act). These requirements also cover a broad range of different, complex ways to define healthcare-associated infections (HCAI), with the standard parameters being infection rates and incidence densities. One entity of HCAI with a high impact for patients, healthcare workers, hospitals and the community are bloodstream infections. In this context, venous catheter (line) associated bloodstream infections (CLABSI) serve as a worldwide accepted benchmark parameter. In addition to this well-established, but laborious to define, parameter, there has been increasing discussion among IPC experts as to whether hospital-onset bacteremia (HOB) is also a suitable measure for surveillance^36,37^. In a subsequent medical informatics, multi-site project (RISK PRINCIPE: risk prediction for IPC)^38^, we will address automated, digital surveillance and prediction of HOB as another perspective of an infection control support system.

Furthermore, in the future it is planned to integrate a new outbreak detection algorithm from our group, which is on par with or better than used state-of the art approaches^39^. Based on weekly case counts from microbiological data, the algorithm generates alarms to inform the user about potential outbreaks for pathogens and wards which are being investigated. The algorithm models the observed number of cases using a hidden Markov model combined with a statistical background model. As the architectural setup in Fig. 1 hints, the outbreak detection algorithm is already planned to be incorporated into the SmICS. An integration of this component into SmICS is waiting for the final phase of the validation. As a next step in translation into routine medical practice, certification as a medical device needs to be assessed. In this way, SmICS’s potential to significantly accelerate the completion of routine tasks that must be performed regularly comes into effect. The IPCT’s capacity that is freed up can then be reinvested into advanced tasks, such as in-depth clarifications, analyzes, and practical actions.

In this article, we present the design, implementation and evaluation of an open-source, openEHR-based application for supporting automated monitoring of bacteria and transmission events and associated epidemiological tasks (called SmICS). Despite the demonstrated feasibility and benefits of developing a standard-based, openEHR application for the IPC domain, broader adoption of open standards and interfaces by software manufacturers is rare. This reluctance is often driven by business strategies favoring proprietary solutions, as well as skepticism regarding the maturity, functionality, and practical value of open data models and associated technologies. Through our work, we aim to showcase the potential and added value of open, interoperable systems for IPC while also openly acknowledging current challenges, such as infrastructural variability impacting usability. Importantly, we also contribute to the broader community by making our newly created, standardized openEHR data models freely available through the Clinical Knowledge Manager, fostering transparency, reuse, and further development within the community. In our work, initial evaluation in an efficiency and usability study based on real-life examples demonstrated that, by using SmICS, some of the most time-consuming basic IPC tasks can be successfully managed in a timely and efficient manner, but SmICS still needs major improvement in terms of usability. Query performance due to varying site-specific infrastructural implementations of the underlying data repository was identified as a remaining issue. When solved, the potential time savings with SmICS will allow IPCTs to use their capacity to focus on verification of outbreaks, advanced analysis and expert-driven intervention and outbreak control tasks in future scenarios. The openEHR-based and open design of SmICS allows roll out, reusability and cross-institutional sharing as well as community-driven enhancements.

## Methods

### Setting

The development of the SmICS software was initiated as a component of the HiGHmed Infection Control project. The HiGHmed Infection Control project brings together various university hospitals in Germany, along with further research institutions, to elaborate digital solutions for IPC21. This project is part of the HiGHmed consortium, which is itself part of the Medical Informatics Initiative (MII) and Research Network of University Medicine (NUM) in Germany. Together, MII and NUM aim to enable the secondary use and sharing of data within and between hospitals. HiGHmed creates an open health data platform facilitating standardization and reuse of data by building MeDIC at each participating institution that is based on a generic and scalable reference architecture for integrating data from care, research, and external sources^20^. For our project, relevant routine data such as microbiology laboratory reports and patient movement data from heterogeneous primary source systems are made interoperable and reusable in each local MeDIC using standardized, agreed-upon semantic and computable data models based on the openEHR standard^40^. In Wulff/Baier et al., we demonstrated the data modeling and integration processes as well as the feasibility of this standardization process in a real-life multicenter setting in full detail^22^. This standardized data basis now facilitates medical data analytics and the development of interoperable applications.

The project was approved by the local ethics committees of the participating sites [Ethics Committee of the Hannover Medical School, no. 9245_BO_K_2020; Ethics Committee of the University Medical Center Göttingen, no. 19/11/18]. All research presented in this manuscript was performed in accordance with applicable relevant guidelines and/or regulations. Appropriate concepts for the use and secure exchange of data were agreed upon with the data protection departments at the respective sites. Informed consent was waived by the ethics committee and the data commissioner [Ethics Committee of the Hannover Medical School, no. 9245_BO_K_2020; Ethics Committee of the University Medical Center Göttingen, no. 19/11/18]. Informed consent is not needed because the study is based on the German Infection Protection Act (‘Infektionsschutzgesetz’, IfSG; German) and the national hygiene regulations (‘Landeshygieneverordnungen’; German). In this article, no individual data points of patients are included.

### Iterative content design and requirements engineering

A flexible, iterative content design process was followed to develop SmICS. Initial requirement analysis was conducted with clinical and technical experts through structured and guided group discussions. Repeated semi-structured interviews and expert dialogues were carried out and prototypes were discussed continuously in tandems. A tandem was formed out of one technical expert and one IPC expert from each partner site. Software development followed an agile, flexible process model. The key architectural demands were that the SmICS should be based solely on clinical routine data, which shall be represented through international and agreed-upon data models and terminologies, and no additional manual data entry is needed. These data shall be analyzed in an appropriate way by using algorithms or thresholding approaches, leading to temporal-spatial visualization. Those shall be used to better understand epidemiological networks and relationships. These features need to be designed as a high-performance system, providing smart support even for rather small and routine everyday tasks. Finally, the SmICS should be freely available under an open-source license.

Over 2 years, we iteratively developed the final visualizations with domain experts from infection control, hygiene management, epidemiology, and related areas. We used quarterly feedback sessions, on-site meetings, and questionnaires to refine multiple prototypes. The early designs of each view were promising but posed challenges for tracing transmissions. A purely “Patient History” view-based approach missed inter-patient contacts, while a “Contact Tracing” view by itself was less intuitive. The “Epidemic Curve” does not show any patient-specific information, and the “Contact Network” itself does not depict any time information. We thus combined the key elements—the “Epidemic Curve”, the “Contact Network”, the “Contact Tracing”, and the “Patient History”—into a final, user-friendly multi-view design for transmission pathway analysis^26^.

### Efficiency and usability evaluation

We evaluated SmICS by comparing time efficiency in performing exemplary tasks of IPC work in a standard procedure versus using SmICS, and by measuring its usability with the SUS^27,39^ and the cSUS.

- Time efficiency: Our aim was to create an application that would support IPCTs in their daily work and strengthen the processes of summarizing and interpreting complex and big data. To test this requirement, three different practical tasks were defined. Professional experience of the IPC specialists involved in the project has shown that these three tasks strongly characterize the everyday life of IPCTs and are often very time-consuming and complex. The tasks involved were the following:

- Task 1: (1.1) Determine how many patients with a positive microbiological finding of a specific Enterobacterales (in any specimen) were treated in a neonatal intensive care unit over a 3-month period. (1.2) Differentiate between hospital and community acquisition of the Enterobacterales for each affected neonate. (1.3) Tabulate the key patient’s data and construct an epidemiological curve. Note: an epidemiological curve is the cornerstone and basic tool for IPC assessment of transmission events/outbreaks.
- Task 2: (2.1) Choose an epidemiological situation, based on the knowledge of a cluster that has taken place, including at least three patients colonized/infected with phenotypically identical MDRB in the same ward. (2.2) Determine whether patients were accommodated in the same room and, if so, for how long. (2.3) Determine the time the patients involved in the cluster have spent together on the ward.
- Task 3: (3.1) Identify the current contact patients of a patient with a newly detected MDRB (colonization or infection). (3.2) Identify patients who have had contact with this MDRB carrier on the same ward during the last 2 weeks and find out if they are still in the hospital and where.

- These tasks would be carried out at as many partner sites as possible with experienced IPCT members using both the standard routine practice and SmICS. In principle, each individual participant (IPCT member) was expected to complete all three tasks, once following the standard procedure and once using SmICS. Two participants at site 1 and four participants at site 3 completed tasks 1–3 only using SmICS. An overview of the participants, along with the time measurements, can be found in the supplementary file. The selection of participants included experienced IPCT members who were available at the time of the evaluation. The standard routine practice included all software tools available in the routine (e.g., hospital or laboratory information systems) and documentation resources that were available at the respective site. Time required for correct completion was measured for each task. The IPCT had no specific training on SmICS, however, a brief guide was provided (written, point-by-point manual). Due to its very simple and structured design, participants were able to complete the tasks on the fly using the manual without specific training in advance. In our interdisciplinary group, we decided that a trained clinician would observe the tasks and check the correctness. To prevent learning effects, each IPC specialist performed each task only once.

- Usability: A usability evaluation was performed using a combined questionnaire (implemented in LimeSurvey Community Edition v5.2.7) comprising the SUS as standardized measure, the cSUS as extension of SUS regarding clinical software, and two free text questions for comments. Bangor et al. showed that a SUS of at least 71.4 counts as “good” usable, 50.9 as “OK” software^27^. The survey had to be filled out by the IPCTs also performing the above-described tasks.

## Supplementary information

Supplementary information

## Data Availability

The datasets supporting the conclusions of this article are included within the article and its additional files. All data models used can be found at https://ckm.highmed.org/ckm. Software components can be found at https://github.com/highmed. Correspondence and requests for materials should be addressed to P.B., C.B. or A.W.

## Abbreviations

AQL: Archetype Query Language
CKM: Clinical Knowledge Manager
CLABSI: Central-Line Associated Bloodstream Infections
COVID-19: Coronavirus 2019 disease
cSUS: Clinical System Usability Scale
HIS: Hospital information system
HOB: Hospital-Onset Bacteremia
IPC: Infection Prevention and Control
IPCT: IPC Teams
HCAI: Healthcare-associated infections
MDRB: Multi-Drug Resistant Bacteria
MeDIC: Medical Data Integration Centers
openEHR: open Electronic Health Record
RISK PRINCIPE: Risk prediction for infection control and treatment in hospitals
SARS-CoV-2: Severe Acute Respiratory Syndrome Coronavirus 2
SmICS: Smart Infection Control System
SUS: System Usability Scale

## Code availability

The code of the SmICS Core and the SmICS Visualization is publicly available at https://github.com/highmed/SmICSCore, https://github.com/highmed/SmICSVisualisierung.
