# Supplementary material for: An openEHR based infection control system to support monitoring of nosocomial bacterial clusters and contacts
Source: NPJ Digit Med. 2025 Jun 30;8:385. doi: 10.1038/s41746-025-01795-9 (PMC12209414; doi:10.1038/s41746-025-01795-9)
Supplement: Supplementary file 1 — Supplementary information [file 41746_2025_1795_MOESM1_ESM.pdf]

An openEHR based infection control system to support monitoring of nosocomial bacterial clusters and contacts

(Biermann/Baier et al.)

Supplementary Information

Content

Detailed statistics for the efficiency study

Usability evaluation of the Smart Infection Control System (SmICS) - Questionnaire

An openEHR based infection control system to support monitoring of nosocomial bacterial clusters and contacts *(Biermann/Baier et al.)*

Measured Times		Site 1 (Entries in min.)							
Task 1	Standard Procedure	30.00	20.00	10.00	15.00	20.00	18		
	Using SmICS	10.00	7.00	8.00	9.00	11.00	14	13	7
Task 2	Standard Procedure	90.00	120.00	80.00	45.00	75.00	100		
	Using SmICS	5.00	5.00	6.00	8.00	7.00	10	11	6
Task 3	Standard Procedure	80.00	110.00	70.00	40.00	80.00	105		
	Using SmICS	12.00	8.00	9.00	11.00	10.00	10	11	8

Measured Times		Site 2 (Entries in min.)					Site 3 (Entries in min.)					
Task 1	Standard Procedure	21.00	17.00	26.00	26.00	26.00	20.00	80.00				
	Using SmICS	18.00	15.00	12.00	17.00	17.00	20.00	6.00	15.00	15.00	5.00	7.00
Task 2	Standard Procedure	25.00	26.00	20.00	17.00	17.00	2.00	2.00				
	Using SmICS	16.00	15.00	13.00	15.00	15.00	15.00	10.00	15.00	15.00	37.00	3.00
Task 3	Standard Procedure	18.00	25.00	21.00	22.00	22.00	250.00	250.00				
	Using SmICS	13.00	15.00	12.00	15.00	15.00	50.00	10.00	45.00	18.00	6.00	18.00

Mean		Site 1					Site 2				
		$\bar{\chi}$ (min)	σ (min)	CI (95%) (min)	$\bar{\chi}_{SP} - \bar{\chi}_{US}$ (min)	Time Saved (%)	$\bar{\chi}$ (min)	σ (min)	CI (95%) (min)	$\bar{\chi}_{SP} - \bar{\chi}_{US}$ (min)	Time Saved (%)
Task 1	Standard Procedure	18.83	6.07	6.37	8.96	47.57	23.20	3.66	4.54	7.40	31.90
	Using SmICS	9.88	2.47	2.07			15.80	2.14	2.65		
Task 2	Standard Procedure	85.00	23.09	24.24	77.75	91.47	21.00	3.85	4.78	6.20	29.52
	Using SmICS	7.25	2.11	1.76			14.80	0.98	1.22		
Task 3	Standard Procedure	80.83	23.17	24.31	70.96	87.78	21.60	2.24	2.79	7.60	35.19
	Using SmICS	9.88	1.36	12.54			14.00	1.26	12.42		
Task 1 - 3	Standard Procedure	61.56	35.84	0.68	52.56	85.38	21.93	3.45	0.70	7.07	32.22
	Using SmICS	9.00	2.38	15.13			14.87	1.71	1.91		

Mean		Site 3					Site 1 - 3				
		$\bar{\chi}$ (min)	σ (min)	CI (95%) (min)	$\bar{\chi}_{SP} - \bar{\chi}_{US}$ (min)	Time Saved (%)	$\bar{\chi}$ (min)	σ (min)	CI (95%) (min)	$\bar{\chi}_{SP} - \bar{\chi}_{US}$ (min)	Time Saved (%)
Task 1	Standard Procedure	50.00	30.00	269.54	38.67	77.33%	**25.31**	16.60	**10.03**	**13.41**	53.00
	Using SmICS	11.33	5.62	5.90			**11.89**	4.42	**2.13**		
Task 2	Standard Procedure	2.00	0.00		-13.83	-691.67%	**47.62**	38.59	**23.32**	**35.67**	74.91
	Using SmICS	15.83	10.40	10.91			**11.95**	7.24	**3.49**		
Task 3	Standard Procedure	250.00	0.00		225.50	90.20%	**84.08**	77.40	**46.77**	**68.50**	81.47
	Using SmICS	24.50	16.87	6.30			**15.58**	11.43	**5.51**		
Task 1 - 3	Standard Procedure	100.67	108.79	17.70	83.44	82.89%	**52.33**	56.32	**18.26**	**39.19**	74.89
	Using SmICS	17.22	13.09	45.94			**13.14**	11.43	**3.03**		

Pooled standard error		Site 1				Site 2			
		$\hat{\sigma}^2$ (min²)	$\hat{\sigma}^2_{inn}$ (min²)	$\hat{\sigma}_E$ (min)	CI (95%) (min)	$\hat{\sigma}^2$ (min²)	$\hat{\sigma}^2_{inn}$ (min²)	$\hat{\sigma}_E$ (min)	CI (95%) (min)
Task 1	Standard Procedure	44.17	22.00	2.53	5.52	16.70	11.20	2.37	5.46
	Using SmICS	6.17				5.70			
Task 2	Standard Procedure	640.00	268.86	8.86	19.29	18.50	9.85	2.22	5.12
	Using SmICS	3.77				1.20			
Task 3	Standard Procedure	644.17	269.57	8.87	19.32	6.30	4.15	1.44	3.32
	Using SmICS	2.00				2.00			
Task 1 - 3	Standard Procedure	1360.03	581.41	7.52	15.20	12.78	7.95	1.07	2.18
	Using SmICS	5.91				3.12			

Pooled standard error		Site 3				Site 1 - 3			
		$\hat{\sigma}^2$ (min²)	$\hat{\sigma}^2_{inn}$ (min²)	$\hat{\sigma}_E$ (min)	CI (95%) (min)	$\hat{\sigma}^2$ (min²)	$\hat{\sigma}^2_{inn}$ (min²)	$\hat{\sigma}_E$ (min)	CI (95%) (min)
Task 1	Standard Procedure	1800.00	331.56	14.87	36.38	298.40	131.75	4.13	**8.44**
	Using SmICS	37.87				20.65			
Task 2	Standard Procedure	0.00	108.14	8.49	20.78	1613.59	678.67	9.38	**19.15**
	Using SmICS	129.77				55.39			
Task 3	Standard Procedure	0.00	284.58	13.77	33.70	6490.58	2678.92	18.63	**38.05**
	Using SmICS	341.50				137.81			
Task 1 - 3	Standard Procedure	14201.07	3367.66	27.36	56.73	3255.65	1358.87	7.66	**15.21**
	Using SmICS	181.36				71.77			

Median		Site 1				Site 2			
		median (min)	minimum (min)	maximum (min)	range (min)	median (min)	minimum (min)	maximum (min)	range (min)
Task 1	Standard Procedure	19.00	10.00	30.00	20.00	26.00	20.00	80.00	60.00
	Using SmICS	10.00	7.00	14.00	7.00	17.00	6.00	20.00	14.00
Task 2	Standard Procedure	85.00	45.00	120.00	75.00	17.00	2.00	20.00	18.00
	Using SmICS	5.00	5.00	11.00	6.00	15.00	10.00	15.00	5.00
Task 3	Standard Procedure	80.00	40.00	110.00	70.00	22.00	21.00	250.00	229.00
	Using SmICS	12.00	8.00	12.00	4.00	15.00	10.00	50.00	40.00
Task 1 - 3	Standard Procedure	72.50	10.00	120.00	110.00	22.00	17.00	26.00	9.00
	Using SmICS	9.00	5.00	14.00	9.00	15.00	12.00	18.00	6.00

Median		Site 3				Site 1 - 3			
		median (min)	minimum (min)	maximum (min)	range (min)	median (min)	minimum (min)	maximum (min)	range (min)
Task 1	Standard Procedure	50.00	20.00	80.00	60.00	20.00	10.00	80.00	70.00
	Using SmICS	11.00	5.00	20.00	15.00	12.00	5.00	20.00	15.00
Task 2	Standard Procedure	2.00	2.00	2.00	0.00	26.00	2.00	120.00	118.00
	Using SmICS	15.00	3.00	37.00	34.00	11.00	3.00	37.00	34.00
Task 3	Standard Procedure	250.00	250.00	250.00	0.00	70.00	18.00	250.00	232.00
	Using SmICS	18.00	6.00	50.00	44.00	12.00	6.00	50.00	44.00
Task 1 - 3	Standard Procedure	50.00	2.00	250.00	248.00	26.00	2.00	250.00	248.00
	Using SmICS	15.00	3.00	50.00	47.00	12.00	3.00	50.00	47.00

$\hat{\sigma}^2$: Estimated population variance
$\hat{\sigma}^2_{inn}$: Pooled Variance
$\hat{\sigma}_E$:Standard error
CI: Confidence Interval

$\bar{\chi}$: Mean
σ: Standarddeviation
$\bar{\chi}_{SP} - \bar{\chi}_{US}$: Mean difference (Time Saved)

The bold highlighted numbers are used in the article:
- $\bar{\chi}$ and the corresponding confidence interval from the 'Mean'-table were used to create the bar chart.
- Statistics mentioned in the text are $\bar{\chi}$SP - $\bar{\chi}$US from the 'Mean'-table and the corresponding confidence interval from the 'Pooled standard error'-table.

Usability evaluation of the Smart Infection Control System (SmICS)

Questionnaire

1. How old are you?
Please choose just one oft he following answers:

- o < 20 years
- o 20 – 30 years
- o 31 – 40 years
- o 41 – 50 years
- o 51– 60 years
- o > 60 years

1. Please name your sex?
Please choose just one oft he following answers:

- o Male
- o Female
- o Divers

3. To which profession do you belong?
Please choose all fitting answers:

- ☐ Hygiene specialist
- ☐ Hygiene officer for nursing staff
- ☐ Nursing staff (without function as hygiene officer / hygiene specialist)
- ☐ Doctor in charge of hygiene
- ☐ Hospital hygienist
- ☐ Specialist in hygiene and environmental medicine
- ☐ Medical doctor in the public health service
- ☐ Resident (without function as hygiene officer)
- ☐ Specialist (without function as hygiene officer)
- ☐ Other: ____________________

4. Where do you work?

Please choose just one of the following answers:

- ○ Berlin
- ○ Göttingen
- ○ Hannover
- ○ Heidelberg
- ○ Lübeck
- ○ Münster
- ○ Other: [ ]

5. How much experience do you have using the Smart Infection Control System?

Please choose just one of the following answers:

- ○ I have used it for the first time
- ○ I used it before
- ○ I used it regulary
- ○ I was involved in the development of the system

6. What did you particularly like about the system?

Please enter your answers here:

7. What did you dislike about the system?

Please enter your answers here:

System Usability Scale (SUS)

Please answer the statements that apply to the System Usability Scale.
Please just choose one answer per statement.

	Strongly disagree 1	2	3	4	Strongly agree 5
I need to learn a lot of things before I could get going with this system.	○	○	○	○	○
I felt very confident using the system.	○	○	○	○	○
I found the system very cumbersome to use.	○	○	○	○	○
I would imagine that most people would learn this system very quickly.	○	○	○	○	○
I thought there was to much inconsistency in this system.	○	○	○	○	○
I found the various functions in this system were well integrated	○	○	○	○	○
I think that I would need the support of a technical person to be able to use this system.	○	○	○	○	○
I thought the system was easy to use.	○	○	○	○	○
I found the system unecessarily complex.	○	○	○	○	○
I think that I would like to use this system frequently.	○	○	○	○	○

Clinical Software Usability Scale

Please answer the statements that apply to the Clinical Software Use.

Please just choose one answer per statement.

	Strongly disagree 1	2	3	4	Strongly agree 5
Using the software gives me the key information I need on patient's history, diagnosed conditions and current care and treatment plan.	○	○	○	○	○
The quality of the interaction/consultation with the patient is adversely affected by the use of this software.	○	○	○	○	○
In my opinion, the software improves the quality of clinical care I can provide.	○	○	○	○	○
Effective support for this software is hard to access in a clinically-appropriate timescale.	○	○	○	○	○
In my opinion, the software reduces the risk of clinical error.	○	○	○	○	○

You can provide additional feedback here:

Please enter your answers here:
